# Epicardial Fat Tissue Volume Within 1 mm of the Left Atrium Combined With Left Atrial Appendage Volume is Associated With Thrombus in Nonvalvular Atrial Fibrillation: A CCTA‐Based Risk Stratification Model

**DOI:** 10.1155/crp/4276622

**Published:** 2026-08-04

**Authors:** Enyuan Zhang, Xiaoyan Wu, Fengmin Lu, Yitong Yin, Shuo Liang, Le He, Henan Zhang, Dongyan Wu, Jing Xu, Qingliang Chen, Wei Ma

**Affiliations:** ^1^ Tianjin Medical University, Tianjin, China, tijmu.edu.cn; ^2^ Heart Rhythm Center, Department of Cardiology, Chest Hospital, Tianjin University, Tianjin, China, tju.edu.cn; ^3^ Department of Pediatric Surgery, General Hospital of Tianjin Medical University, Tianjin, China, tjmugh.com.cn; ^4^ Department of Radiology, Chest Hospital, Tianjin University, Tianjin, China, tju.edu.cn; ^5^ Department of Cardiac Surgery, Chest Hospital, Tianjin University, Tianjin, China, tju.edu.cn

**Keywords:** atrial fibrillation, computed tomography angiography, epicardial fat tissue, left atrial appendage thrombus, nomogram, risk prediction

## Abstract

**Background:**

Accurate prediction of left atrial appendage thrombus (LAAT) is critical for stroke prevention in patients with nonvalvular atrial fibrillation (AF). We investigated the incremental value of epicardial fat tissue (EFT) metrics derived from cardiac computed tomography angiography (CCTA) beyond conventional risk factors.

**Methods:**

In this retrospective study of 287 AF patients prior to ablation, CCTA was used to measure left atrial appendage volume (LAAV) and EFT volume within 1 mm of the left atrium (EFT1). Multivariate logistic regression analysis identified predictors of LAAT or prethrombotic state (thrombus/spontaneous echo contrast), which were incorporated into a nomogram. Model performance was evaluated using the area under the curve (AUC), calibration plots, and decision curve analysis.

**Results:**

The thrombus group exhibited significantly larger LAAV (15.07 ± 2.7 mL vs. 11.94 ± 2.55 mL, *p* < 0.001) and EFT1 volume (6.39 ± 1.38 mL vs. 5.36 ± 1.47 mL, *p* < 0.001) compared to the normal/SEC group. Multivariate analysis identified EFT1 (odds ratio [OR] = 1.57 per mL), LAAV (OR = 1.56 per mL), and the CHA_2_DS_2_‐VA score (OR = 1.34 per point) as independent predictors. The resulting nomogram demonstrated excellent discrimination for LAAT (AUC = 0.851) and for thrombus/SEC (AUC = 0.819), with good calibration and clinical utility.

**Conclusion:**

EFT1, the EFT volume immediately adjacent to the left atrium, is strongly associated with LAAT. The novel nomogram integrating EFT1, LAAV, and the CHA_2_DS_2_‐VA score provides enhanced risk stratification for thrombotic and prethrombotic states in patients with AF.

## 1. Introduction

Left atrial appendage thrombus (LAAT) is a critical source of thromboembolism in patients with atrial fibrillation (AF) or atrial flutter (AFL), accounting for up to 90% of AF‐related strokes [1]. Despite guideline‐directed anticoagulation, the prevalence of LAAT remains significant (4.3%–23%), particularly in high‐risk subgroups [1, 2]. Current risk stratification tools, such as the CHA_2_DS_2_‐VA score, demonstrate limited discrimination for LAAT (area under the curve = 0.619), underscoring the need for more refined prediction models [1, 3]. Emerging evidence highlights the value of integrating clinical, echocardiographic, and biochemical markers to improve LAAT risk assessment [3–6].

The evaluation of left atrial appendage (LAA) thrombus is critical in patients with AF to prevent thromboembolic events. While transesophageal echocardiography (TEE) has traditionally been the gold standard, recent evidence demonstrates that cardiac computed tomography angiography (CCTA) offers superior effectiveness and accuracy in detecting LAA thrombus and distinguishing it from spontaneous echo contrast (SEC). A meta‐analysis revealed that CCTA with delayed imaging achieved a sensitivity of 96% and specificity of 92% for LAA thrombus detection, which improved to 99% specificity and 92% positive predictive value with delayed phases, outperforming TEE in ruling out thrombus [7]. This is further supported by another study that found a CCTA protocol incorporating a 6‐min delayed phase achieved 100% specificity and positive predictive value, effectively eliminating false positives caused by SEC [8]. Additionally, dual‐energy CCTA techniques demonstrated higher diagnostic accuracy than conventional Hounsfield unit (HU) measurements, with iodine concentration and effective atomic number (Z_eff) ratios providing better differentiation between thrombus and SEC [9]. A network meta‐analysis confirmed that dual‐source CCTA (DSCTA) had the highest sensitivity (71.7%) and specificity (84.3%) among imaging modalities, surpassing TEE [10]. In clinical practice, Bilchick et al. [11] demonstrated that integrating delayed CCTA imaging into preablation care reduced the need for TEE from 57.5% to 24.0% without increasing stroke risk, highlighting its feasibility and safety. Moreover, Palmer et al. [12] reported 100% sensitivity and 98% specificity for CCTA in detecting LAA thrombus in patients undergoing transcatheter aortic valve replacement, with no false negatives. Collectively, these findings indicate that CCTA, particularly with advanced protocols, is more effective and accurate than TEE for LAA thrombus assessment and SEC differentiation, offering a non‐invasive, efficient alternative that enhances patient care.

Therefore, this study aimed to investigate whether novel parameters derived from CCTA, specifically left atrial appendage volume (LAAV) and epicardial adipose tissue volume within 1 mm of the left atrium (EFT1), could be combined with the conventional CHA_2_DS_2_‐VA score to develop a predictive model for LAAT or prethrombotic state (SEC) in patients with nonvalvular AF. We hypothesized that such a model could serve as a supplementary tool for preprocedural risk stratification, potentially enabling a more targeted use of TEE.

## 2. Methods

### 2.1. Study Population and Cohort Stratification

**FIGURE 1:**
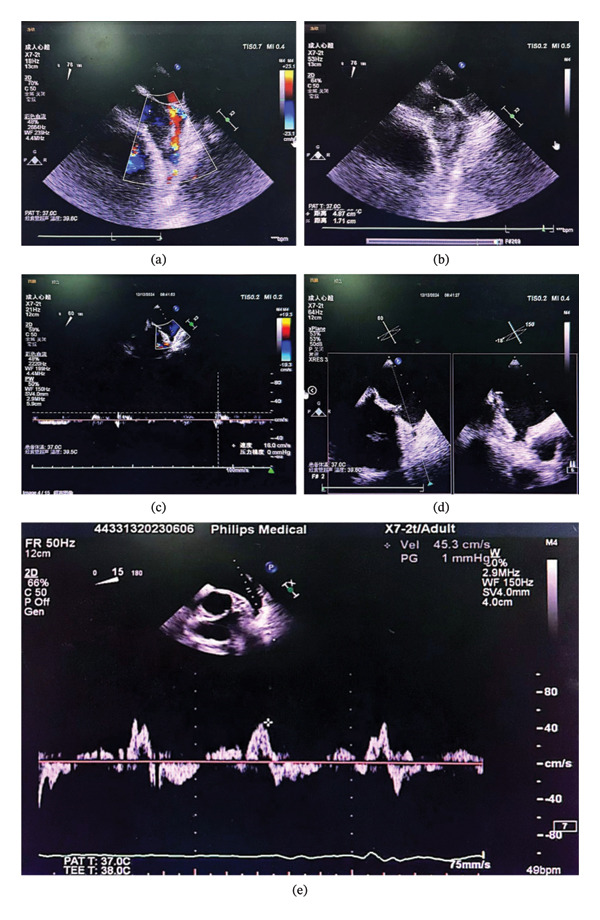
(a, b) TEE images showing a solid, echo‐dense mass. (c, d) TEE images showing dynamic, smoke‐like echoes within the LAA. (e) TEE image of a normal LAA without significant SEC.

We conducted a single‐center, retrospective cohort study of consecutive adults (aged ≥ 18 years) with AF or atypical AFL scheduled for catheter ablation between January 2018 and September 2025. Inclusion criteria are as follows: (1) patients diagnosed with AF have received anticoagulation for at least 2 weeks; (2) preprocedural CCTA reports indicating “indeterminate for LAAT poor contrast opacification or motion artifact; and (3) planned TEE for definitive LAA evaluation within 48 h after CCTA. To ensure measurement reliability, CCTA scans initially reported as “indeterminate for LAAT” due to suboptimal contrast opacification or motion artifact were retrospectively re‐evaluated by two experienced radiologists. Only scans in which the left atrial border and pericardial fat could be clearly delineated for volumetric analysis were included. Scans with artifacts that obscured the pericardial fat boundary or LAA ostium were excluded from the final analysis. Exclusion criteria include (1) contraindications to TEE or anticoagulation and (2) documented hypercoagulable states unrelated to AF or AFL (e.g., antiphospholipid syndrome and active malignancy). Based on TEE findings, patients were stratified into three groups: Group 1 (definite LAA thrombus, Figure 1a,b), Group 2 (prethrombotic state/SEC: moderate to severe SEC with or without LAA emptying velocity [LAAEV] ≤ 25 cm/s, Figure 1c,d), and Group 3 (normal: no SEC or mild SEC with LAAEV > 40 cm/s, Figure 1e). To address distinct clinical questions, two primary analyses were performed: (1) The Thrombus Analysis Cohort: To evaluate predictors of definite LAAT, we compared the “Definite Thrombus” group (Group 1) versus the “No Thrombus” group (combined Groups 2 and 3, i.e., ‘Normal + SEC’). (2) The Hypercoagulable State Analysis Cohort: To evaluate predictors of any hypercoagulable state (including both thrombus and SEC), we compared the “Thrombus/SEC” group (combined Groups 1 and 2) versus the “Normal” group (Group 3). The rationale for sludge and LAAEV thresholds is based on two previous studies [3, 13].

This retrospective study was conducted in accordance with the Declaration of Helsinki and was approved by the Ethics Committee of Tianjin Chest Hospital (Approval Number: 2026W‐019). The requirement for informed consent was waived due to the retrospective design.

### 2.2. Data Collection

Extract baseline clinical information from the electronic medical record system, including gender, age, type of AF (paroxysmal or continuous), duration of AF or palpitations, presence or absence of concomitant AFL, clinical comorbidities (hypertension, diabetes, coronary heart disease, and cerebral infarction), and echocardiographic parameters (anteroposterior diameter of the left atrium, end‐diastolic diameter of the left ventricle, left‐right diameter of the right atrium, left ventricular ejection fraction, moderate or greater mitral, tricuspid, or aortic valve regurgitation, and LAAEV measured during TEE). The CHA2DS2‐VA score was also calculated based on the clinical condition of each patient. Laboratory indicators included D‐dimer (μg/mL), creatinine level (μmol/L), and B‐type natriuretic peptide (pg/mL). Anticoagulation regimen was recorded, including the proportion of patients on non‐vitamin K antagonist oral anticoagulants (NOACs) versus vitamin K antagonists (VKAs). For patients on VKAs, time in therapeutic range (TTR) was calculated using the Rosendaal method.

### 2.3. CCTA Imaging and Analysis

**FIGURE 2:**
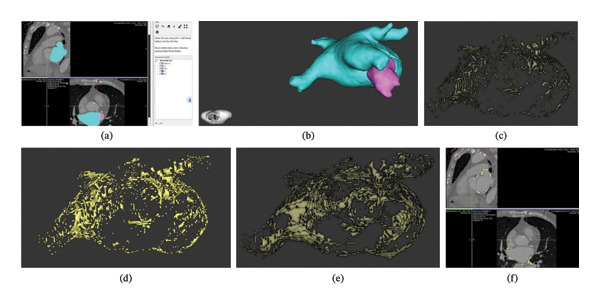
(a, b) 3D reconstructions for measuring left atrial (LA) and left atrial appendage (LAA) volumes. (c) Cross‐sectional CT image showing fat infiltration within the LA wall. (d) 3D reconstruction showing epicardial adipose tissue volume in a 1‐mm shell around the LA. (e, f) Images illustrating epicardial fat measurement at 3‐mm distances from the LA surface.

All patients underwent LA‐CCTA examination using a 320‐slice CT scanner. Using ADAS image postprocessing software, the following parameters were extracted: left atrial sphericity (LAS), calculated by the formula [1 − (actual LA volume/minimum external ball volume)] × 100%; LA volume (mL) and area (cm^2^); and LAA volume (mL) and area (cm^2^), Figure 2a,b. Fat parameters were defined based on CT value thresholds: the CT value range for fat was from −194 HU to −30 HU and for dense fat, from −194 HU to −50 HU. The calculated indicators included LA myocardium infiltration fat volume (inFAT, mL, Figure 2c); epicardial fat volume (EFT‐1/Figure 2d, EFT‐3/Figure 2e,f, EFT‐5, EFT‐10, mL), measured as the volume radiating outward from the left atrium by 1 mm, 3 mm, 5 mm, and 10 mm, respectively; dense inFAT volume (Dense inFAT, mL); and dense EFT‐1 volume (mL). All measurements were independently performed by two experienced radiologists, with any discrepancies resolved by consensus.

Quantification of EFT1 volume: EFT1 measurement was performed semiautomatically using dedicated postprocessing software (ADAS). The steps were as follows: (a) The LA and LAA were first segmented using the “LA WALL THICKNESS” and “HEART ANATOMY EXTRACTION” modules. (b) The “Dilate Cardiac Model” function was then applied to the segmented LA model with a dilation value set to 1 mm, creating a 3D shell extending 1 mm outward from the LA epicardial surface. (c) The software automatically calculated the total volume of all voxels within this 1‐mm shell that fell within the predefined fat HU range (−194–−30 HU), which was defined as EFT1.

Reproducibility analysis: To assess the interobserver reliability of CCTA measurements, a subset of studies was independently analyzed by two experienced radiologists who were blinded to each other’s results and the patients’ data.

### 2.4. TEE Protocol

TEE will be performed by experienced operators blinded to CCTA results, following a standardized protocol. LAA visualization will include more than 20 frames from multiplane angles (0°, 45°, 90°, and 135°), incorporating Doppler‐based LAAEV measurements [13]. LAAT is defined as a solid, circumscribed, echo‐dense mass with independent motion, confirmed in two or more planes [14]. LAA sludge, or prethrombotic state, is characterized by dynamic, smoke‐like SEC without a discrete mass; it is graded as mild (barely detectable) or severe (smoke obscuring anatomy) according to the Huang SEC scale [15].

### 2.5. Statistical Analysis

The analysis included measures such as the mean (standard deviation) for continuous variables and frequencies with percentages for categorical variables. Group comparisons for continuous variables were conducted using Welch’s *t*‐test or ANOVA. For comparisons between groups involving categorical data, the Fisher exact test was used when expected frequencies were less than 5; otherwise, the chi‐squared test was applied. Interobserver agreement was quantified using the intraclass correlation coefficient (ICC) for absolute agreement based on a two‐way random‐effects model. ICC values were interpreted as follows: < 0.50, poor; 0.50–0.75, moderate; 0.75–0.90, good; and > 0.90, excellent reliability. Bland–Altman analysis was performed to calculate the mean difference (bias) and 95% limits of agreement (LoA) between observers. To minimize overfitting and enhance model generalizability, we used penalized logistic regression with the Least Absolute Shrinkage and Selection Operator (LASSO) for variable selection and coefficient estimation. LASSO applies an L1 penalty to shrink less relevant coefficients toward zero, effectively performing variable selection while accounting for potential multicollinearity among predictors. The optimal penalty parameter (λ) was determined via 10‐fold cross‐validation to minimize the deviance. Variables with nonzero coefficients at the optimal *λ* were retained in the final prediction model. Model performance was evaluated using the area under the curve (AUC), calibration plots, and decision curve analysis. To minimize overfitting and enhance model generalizability, we used penalized logistic regression with the LASSO for variable selection and coefficient estimation. LASSO applies an L1 penalty to shrink less relevant coefficients toward zero, effectively performing variable selection while accounting for potential multicollinearity among predictors. The optimal penalty parameter (λ) was determined via 10‐fold cross‐validation to minimize the deviance. Variables with nonzero coefficients at the optimal *λ* were retained in the final prediction model. Model performance was evaluated using AUC, calibration plots, and decision curve analysis. A nomogram prediction model for the risk of thrombus or thrombus/SEC in patients with AF was constructed using R software. The receiver operating characteristic (ROC) curve was used to evaluate the model’s discrimination ability, and a calibration curve was generated to assess the model’s fit. Additionally, a decision curve analysis was performed to evaluate the clinical utility of the nomogram. All statistical analyses in this study were conducted using SPSS (Version 29.0.2.0) and RStudio (Version 9.5.191.52).

## 3. Results

### 3.1. Comparison of Clinical Data Between the Two Groups of Patients

Table 1 presents the baseline characteristics of the study population stratified by the LAA status. The cohort included 202 patients in the no thrombus group and 85 in the thrombus group. There were no significant differences between the groups in age (66 ± 9 vs. 66 ± 10 years, *p* = 0.527), gender distribution (male: 67.8% vs. 67.1%, *p* = 0.900), AF type (persistent: 73.3% vs. 77.6%, *p* = 0.437), AFL (15.8% vs. 20.0%, *p* = 0.393), AF history duration (56 ± 82 vs. 81 ± 111 months, *p* = 0.065), hypertension (60.9% vs. 64.7%, *p* = 0.543), diabetes (22.8% vs. 17.6%, *p* = 0.333), PCI history (30.7% vs. 32.9%, *p* = 0.708), CHD (39.1% vs. 41.2%, *p* = 0.744), D‐dimer (0.76 ± 0.63 vs. 0.77 ± 0.45 mg/L, *p* = 0.889), BNP (253 ± 144 vs. 255 ± 133 pmol/L, *p* = 0.918), or creatinine levels (90 ± 33 vs. 87 ± 34 μmol/L, *p* = 0.525). However, the thrombus group had significantly higher CHA_2_DS_2_‐VA score compared to the no thrombus group (2.36 ± 1.10 vs. 2.06 ± 1.16, *p* = 0.018).

**TABLE 1:** Baseline characteristics of patients with and without left atrial appendage thrombus.

	LAA status			
Characteristic	No thrombus^†^ *N* = 202	Thrombus *N* = 85	Statistic	*p* value
Age, Mean ± SD	66 ± 9	66 ± 10	−0.63	0.527
GENDER, *n* (%)			0.02	0.900
Female	65 (32.2%)	28 (32.9%)		
Male	137 (67.8%)	57 (67.1%)		
AF TYPE, *n* (%)			0.61	0.437
Paroxymal	54 (26.7%)	19 (22.4%)		
Persistent	148 (73.3%)	66 (77.6%)		
AFL, *n* (%)			0.73	0.393
No	170 (84.2%)	68 (80.0%)		
Yes	32 (15.8%)	17 (20.0%)		
AF history (months), Mean ± SD	56 ± 82	81 ± 111	−1.86	0.065
HTN, *n* (%)			0.37	0.543
No	79 (39.1%)	30 (35.3%)		
Yes	123 (60.9%)	55 (64.7%)		
DM, *n* (%)			0.94	0.333
No	156 (77.2%)	70 (82.4%)		
Yes	46 (22.8%)	15 (17.6%)		
PCI, *n* (%)			0.14	0.708
No	140 (69.3%)	57 (67.1%)		
Yes	62 (30.7%)	28 (32.9%)		
CHD, *n* (%)			0.11	0.744
No	123 (60.9%)	50 (58.8%)		
Yes	79 (39.1%)	35 (41.2%)		
CHA2DS‐VA, Mean ± SD	2.00 ± 1.20	2.38 ± 1.16	2.48	0.014
D‐dimer (mg/L), Mean ± SD	0.76 ± 0.63	0.77 ± 0.45	−0.14	0.889
BNP (pmol/L), Mean ± SD	253 ± 144	255 ± 133	−0.10	0.918
Creatinine (μmol/L), Mean ± SD	90 ± 33	87 ± 34	0.64	0.525
Anticoagulation regimen				
NOACs (%)	94.1	91.8	0.12	0.729
VKAs (%)	5.9	8.2		
TTR for VKAs (mean %)	68.4 ± 12.1	65.2 ± 14.3	1.58	0.114

*Note:* AFL = atrial flutter; HTN = hypertension; LAAD = left atrial appendage; NOAC = novel oral anticoagulants.

Abbreviations: AF, atrial fibrillation; BNP, B‐type natriuretic peptide; CHD, coronary heart disease; DM, diabetes mellitus; PCI, previous cerebral infarction; SEC, spontaneous echo contrast; TTR, time in therapeutic range; VKA, Vitamin K antagonists.

^†^The “No Thrombus” group includes patients with a normal TEE and those with spontaneous echo contrast (SEC).

Table 2 presents cardiac ultrasound and CCTA‐measured indicators for the two groups based on the LAA status. The thrombus group exhibited significantly larger left atrial volume (LAV: 207 ± 56 mL vs. 192 ± 59 mL, *p* = 0.043), LAAV (15.07 ± 2.7 mL vs. 11.94 ± 2.55 mL, *p* < 0.001), and LAA area (37 ± 14 cm^2^ vs. 34 ± 13 cm^2^, *p* = 0.042) compared to the no thrombus group. Significant differences were also observed in EFT1 measurements across both attenuation ranges (−194 to −30 HU: 6.39 ± 1.38 mL vs. 5.36 ± 1.47 mL, *p* < 0.001; −194 to −50 HU: 3.77 ± 3.31 mL vs. 2.80 ± 1.90 mL, *p* = 0.013). No statistically significant differences were found in other echocardiographic parameters, valvular regurgitation prevalence, or remaining EFT measurements between groups (all *p* > 0.05).

**TABLE 2:** Cardiac ultrasound and cCTA‐measured indicators of the two groups.

	LAA status			
**Characteristic**	**Normal/SEC *N* = 202**	**Thrombus *N* = 85**	**Statistic** ^1^	***p* value** ^1^
LA (mm), Mean ± SD	45 ± 7	46 ± 6	−1.70	0.091
LV (mm), Mean ± SD	51 ± 6	53 ± 8	−1.42	0.159
RA (mm), Mean ± SD	43 ± 7	43 ± 7	−0.33	0.743
PAP (mmHg), Mean ± SD	33.5 ± 6.8	34.2 ± 6.5	−0.83	0.408
LVEF (%), Mean ± SD	55 ± 9	53 ± 11	1.83	0.069
AR, *n* (%)			0.27	0.601
No	178 (88.1%)	73 (85.9%)		
Yes	24 (11.9%)	12 (14.1%)		
MR, *n* (%)			0.02	0.894
No	130 (64.4%)	54 (63.5%)		
Yes	72 (35.6%)	31 (36.5%)		
TR, *n* (%)			0.16	0.685
No	128 (63.4%)	56 (65.9%)		
Yes	74 (36.6%)	29 (34.1%)		
LAS (%), Mean ± SD	78.5 ± 12.9	77.1 ± 3.7	0.59	0.561
LAV (ml), Mean ± SD	192 ± 59	207 ± 56	−2.04	0.043
LA area (cm^2^), Mean ± SD	220 ± 48	228 ± 35	−1.52	0.129
LAAV (ml), Mean ± SD	11.94 ± 2.55	15.07 ± 2.7	−9.11	< 0.001
LAA area (cm^2^), Mean ± SD	34 ± 13	37 ± 14	−2.06	0.042
AT (mm), Mean ± SD	1.32 ± 0.15	1.31 ± 0.13	0.43	0.669
inFAT (−194 ⟶ −30, ml), Mean ± SD	1.47 ± 0.61	1.63 ± 0.67	−1.83	0.069
inFAT (−194 ⟶ −50, ml), Mean ± SD	0.78 ± 0.51	0.89 ± 0.49	−1.68	0.094
EFT1 (−194 ⟶ −30, ml), Mean ± SD	5.36 ± 1.47	6.39 ± 1.38	−5.66	< 0.001
EFT1 (−194 ⟶ −50, ml), Mean ± SD	2.80 ± 1.90	3.77 ± 3.31	−2.51	0.013
EFT3 (−194 ⟶ −50, ml), Mean ± SD	14 ± 7	15 ± 8	−1.38	0.171
EFT5 (−194 ⟶ −50, ml), Mean ± SD	21 ± 10	23 ± 12	−1.30	0.197
EFT10 (−194 ⟶ −50, ml), Mean ± SD	36 ± 17	39 ± 21	−1.34	0.184

Abbreviations: AR, aortic regurgitation (moderate or above); AT, average thickness; cCTA, cardiac computerized tomography angiography; EFT, epicardial fat tissue; inFAT, intramyocardial fat; LA, left atrium; LAA, left atrial appendage; LAAV, left atrial appendage volume; LAS, left atrial sphericity; LAV, left atrial volume; LV, left ventricle; LVEF, left ventricular ejection fraction; MR, mitral regurgitation (moderate or above); PAP, pulmonary artery pressure; RA, right atrium; SEC, spontaneous echo contrast; TR, tricuspid regurgitation (moderate or above).

The interobserver reliability for all CCTA‐measured parameters was excellent, as detailed in Table 3. The interobserver agreement for volumetric and fat measurements derived from CCTA was consistently high. Key structural parameters such as LAV (ICC = 0.97) and LAAV (ICC = 0.95) demonstrated near‐perfect reliability, which is critical for their use in risk stratification models. The epicardial adipose tissue measurements, which are central to this study’s hypothesis, also showed excellent reproducibility. Specifically, the volume of epicardial fat within a 1‐mm shell surrounding the left atrium (EFT1) had an ICC of 0.92, indicating highly consistent measurements between independent observers. The Bland–Altman analysis for EFT1 revealed a minimal mean bias of 0.15 mL, with 95% LoA from −0.45 mL to 0.75 mL, suggesting that repeated measurements are unlikely to differ by more than approximately 0.75 mL in a clinical setting. The reliability for fat measurements using the denser attenuation threshold (−194 to −50 HU) was similarly excellent (ICC for Dense EFT1 = 0.91). The high reproducibility observed across all metrics confirms that the CCTA measurement protocol used in this study is robust and that the parameters (particularly EFT1 and LAAV) are suitable for use in subsequent predictive modeling.

**TABLE 3:** Interobserver reliability of CCTA parameters.

Parameter	ICC (95% CI)	Mean difference (Observer 1–Observer 2)	95% limits of agreement
Left atrial/appendage metrics			
LAS (%)	0.94 (0.91–0.96)	−0.15%	–2.8%–2.5%
LAV (mL)	0.97 (0.95–0.98)	1.2 mL	–8.5–10.9 mL
LA Area (cm^2^)	0.96 (0.94–0.97)	0.3 cm^2^	–3.1–3.7 cm^2^
LAAV (mL)	0.95 (0.93–0.97)	0.4 mL	–2.1–2.9 mL
LAA area (cm^2^)	0.93 (0.90–0.95)	0.5 cm^2^	–3.0–4.0 cm^2^
AT (mm)	0.91 (0.87–0.94)	0.05 mm	–0.35 to 0.45 mm
Fat metrics (−194–−30 HU)			
inFAT (mL)	0.92 (0.89–0.94)	0.08 mL	–0.95–1.11 mL
EFT1 (mL)	0.92 (0.88–0.95)	0.15 mL	–0.45–0.75 mL
EFT3 (mL)	0.94 (0.91–0.96)	0.22 mL	–1.10–1.54 mL
EFT5 (mL)	0.95 (0.93–0.97)	0.18 mL	–1.85–2.21 mL
EFT10 (mL)	0.96 (0.94–0.97)	0.25 mL	–2.50–3.00 mL
Dense fat metrics (−194–−50 HU)			
Dense inFAT (mL)	0.90 (0.86–0.93)	0.05 mL	–0.60–0.70 mL
Dense EFT1 (mL)	0.91 (0.87–0.94)	0.10 mL	–0.50–0.70 mL

### 3.2. Factors Influencing Thrombus Formation in AF Patients

**FIGURE 3:**
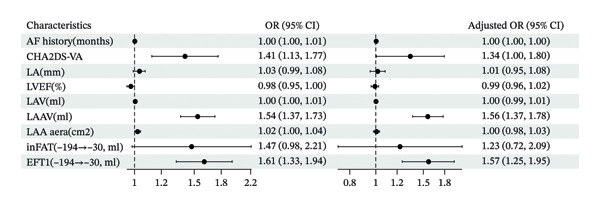
Univariate and multivariate analyses of influencing factors for thrombus formation (logistic regression).

In the multivariate regression model, several continuous variables were significantly associated with LAA status. A one‐unit increase in the CHA2DS‐VA score was associated with higher odds of LAA status (adjusted odds ratio [OR)], 1.34; 95% confidence interval [CI], 1.00–1.80; *p* = 0.047). Similarly, each unit increase in LAAV was associated with increased odds (adjusted OR, 1.56; 95% CI, 1.37–1.78; *p* < 0.001). A unit increase in the EFT1 volume was also associated with higher odds of LAA status (adjusted OR, 1.57; 95% CI, 1.25–1.95; *p* < 0.001; Table 4 and Figure 3). All three variables demonstrated modest but statistically significant effect sizes, with ORs slightly above 1, indicating a positive association with the outcome. The model exhibited acceptable discriminative capacity, with all predictors contributing independently to outcome prediction.

**TABLE 4:** Univariate and multivariate analyses of influencing factors (Logistic regression).

Characteristic	Univariable					Multivariable				
	*N*	Event *N*	OR	95% CI	*p* value	*N*	Event N	OR	95% CI	*p* value
AF history (months)	287	85	1.00	1.00, 1.01	0.044^∗^					
CHA2DS‐VA	287	85	1.41	1.13, 1.77	0.003^∗∗^	287	85	1.34	1.00, 1.80	0.047^∗^
LA (mm)	287	85	1.03	0.99, 1.08	0.107					
LVEF (%)	287	85	0.98	0.95, 1.00	0.047^∗^					
LAV (ml)	287	85	1.00	1.00, 1.01	0.049^∗^					
LAAV (ml)	287	85	1.54	1.37, 1.73	< 0.001^∗∗∗^	287	85	1.56	1.37, 1.78	< 0.001^∗∗∗^
LAA area (cm^2^)	287	85	1.02	1.00, 1.04	0.034^∗^					
inFAT (−194 ⟶ −30, ml)	287	85	1.47	0.98, 2.21	0.060					
EFT1 (−194 ⟶ −30, ml)	287	85	1.61	1.33, 1.94	< 0.001^∗∗∗^	287	85	1.57	1.25, 1.95	< 0.001^∗∗∗^

*Note:* Null deviance = 349; Null df = 286; Log‐likelihood = −123; AIC = 265; BIC = 302; Deviance = 245; Residual df = 277; No. Obs. = 287.

Abbreviations: CI, confidence interval; OR, odds ratio.

^∗^ *p* < 0.05.

^∗∗^ *p* < 0.01.

^∗∗∗^ *p* < 0.001.

### 3.3. Construction of the Risk Nomogram Model

**FIGURE 4:**
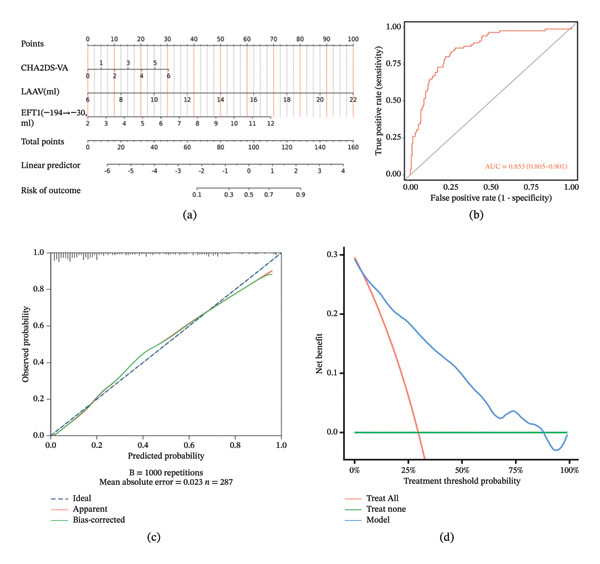
(a) The nomogram for predicting thrombus developed based on the training cohort. (b) Receiver operating characteristic (ROC) curve of the prediction thrombus. (c) Calibration curve of nomogram for thrombus. (d) DCA curve of nomogram for thrombus.

Based on the results of the logistic regression analysis, CHA2DS‐VA, LAAV, and EFT1 were identified as significant factors influencing LAA thrombosis in patients with AF. The logistic regression model is expressed as follows: Logit(P) = −10.439 + 0.44 (LAAV, ml) + 0.485 (EFT1 volume, ml) + 0.357 (CHA2DS‐VA). Using these predictor variables, a nomogram was developed to predict LAAT formation in AF patients. The results showed that for every 1‐point increase in the CHA2DS‐VA score, the model score increases by 5 points. For every 1 mL increase in EFT1 above 2 mL, the model score increases by 7 points. For every 1 mL increase in LAAV above 6 mL, the model score increases by 6 points (Figure 4a).

### 3.4. Verification of the Risk‐Prediction Nomogram Model

The proposed predictive model demonstrated robust performance in the internal cohort, achieving an AUC of 0.853 (95% CI: 0.805–0.901). These results indicate strong discriminative ability and suggest that the model holds promise for clinical application. However, further validation in external populations is warranted to confirm its generalizability, as illustrated in Figure 4b. Internal validation used 10‐fold cross‐validation to evaluate the model’s robustness and discriminative ability. The internally validated C‐index/AUC was 0.844 (95% CI 0.796–0.893). Figure 4c shows a good fit of the nomogram model, as demonstrated by the calibration curve. Additionally, decision curve analysis results, presented in Figure 4d, reveal that the clinical net benefit of this nomogram model is greater than zero.

### 3.5. The Prediction of Thrombus/SEC by Above Indicators

**FIGURE 5:**
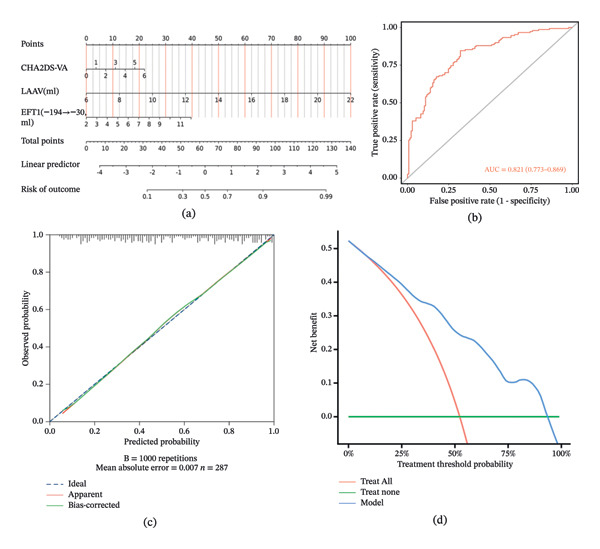
(a) The nomogram for predicting thrombus/SEC developed based on the training cohort. (b) Receiver operating characteristic (ROC) curve of the prediction thrombus/SEC. (c) Calibration curve of nomogram for thrombus/SEC. (d) DCA curve of nomogram for thrombus/SEC.

When thrombus and SEC were used as outcome variables, the nomogram reconstructed using the aforementioned indicators continued to demonstrate strong predictive capability. The logistic regression model is expressed as follows: Logit(P) = −10.439 + 0.44 × (LAAV, ml) + 0.485 × (EFT1 volume, ml) + 0.357 × (CHA2DS‐VA score). Using these predictor variables, a nomogram was developed to predict LAAT formation in patients with AF. The results showed that for every 1‐point increase in the CHA2DS‐VA score, the model score increases by 3.8 points. For every 1‐mL increase in the EFT1 volume above 2 mL, the model score increases by 4 points. For every 1‐mL increase in LAAV above 6 mL, the model score increases by 6 points (Figure 5a). The prediction model demonstrated robust discriminative ability in the internal cohort, achieving an AUC of 0.821 (95% CI: 0.773–0.869), indicating strong predictive performance and suggesting good generalizability within the development dataset (Figure 5b). Internal validation used 10‐fold cross‐validation to evaluate the model’s robustness and discriminative ability. The internally validated C‐index/AUC was 0.807 (95% CI 0.757–0.857). The calibration curve (Figure 5c) indicates a good fit of the nomogram model. Finally, decision curve analysis (Figure 5d) revealed that the clinical net benefit of this nomogram model is greater than zero. These results indicate that the predictive model, jointly established by LAAV, EFT1 volume, and CHA2DS‐VA score, can not only predict the risk of thrombosis in patients with AF but also stratify and predict the pre‐thrombotic state, namely, SEC.

## 4. Discussion

Our research indicates that local adipose tissue within a 1‐mm radius around the left atrium is more closely associated with thrombosis. Furthermore, in patients with AF, LAAV may be a better predictor of thrombosis than LA volume. Combining these two indicators with the traditional CHA2DS2‐VA score, which reflects the clinical condition, further enhances the ability to predict thrombosis. Additionally, this combined approach shows strong predictive value for prethrombotic hypercoagulable states such as SEC, enabling more effective screening of false‐negative cases and significantly reducing reliance on TEE.

The nomogram developed in this study is intended as a supplementary and predictive tool, not a diagnostic substitute for TEE. In clinical practice, it could identify patients at higher predicted risk who should then proceed to definitive TEE evaluation. This positions CCTA‐derived parameters as “risk enhancers,” aligning with the concept of integrating CCTA to refine imaging pathways and reduce unnecessary TEE examinations.

This study demonstrates that EFT volume, measured by CCTA, is a strong and independent predictor of LAAT formation and cardioembolic stroke. Beyond serving as passive fat storage, EFT is a metabolically active organ that secretes proinflammatory cytokines (e.g., IL‐6 and TNF‐α) and prothrombotic mediators (e.g., PAI‐1 and tissue factor), promoting endothelial dysfunction and a prothrombotic state [16, 17]. Its anatomical proximity to the LAA facilitates direct pathological interaction, as evidenced by a 43.97‐fold increase in LAAT risk among mitral stenosis patients with EFT thickness greater than 4.05 mm [18], along with a dose‐dependent correlation between EFT volume and thrombus density.

The pathophysiological mechanisms linking EFT volume to cerebrovascular events are threefold: (1) Direct thrombogenesis: EFT promotes coagulation by releasing tissue factor and inhibits fibrinolysis through plasminogen activator inhibitor‐1 (PAI‐1), facilitating fibrin deposition independent of blood stasis [19, 20]; (2) Arrhythmogenesis: Paracrine factors secreted by EFT promote atrial fibrosis, creating a substrate for AF—a major cause of cardioembolic stroke [21, 22]; and (3) Impaired cardiac function: EFT volume inversely correlates with LAAEV. Each 5‐mm increase in EFT thickness predicts an 18% decline in LAAEV, supporting the diagnostic value of EFT thickness greater than 5.1 mm for identifying low flow (AUC = 0.83) [13].

EFT thickness correlates with thromboembolic risk as measured by the CHA_2_DS_2_‐VA score in AF. Importantly, multivariate models confirm that an EFT volume ≥ 105 cm^3^ confers a 5.8‐fold increased risk of stroke, independent of AF burden [23]. Deep learning‐based quantification of EFT volume predicts incident stroke, with a hazard ratio (HR) of 1.20 per 10 cm^3^ increase [24]. The clinical impact is clear: patients with high periatrial EFT volume (> 20.15 mL) have significantly reduced stroke‐free survival after AF ablation [25], Additionally, an EFT volume > 112 cm^3^ independently predicts hemorrhagic transformation following thrombolysis (OR = 11.12) [26].

CCTA serves as an ideal platform for EFT volumetry, enabling comprehensive risk stratification. Its integration into clinical pathways can reduce the need for TEE by 58% without compromising stroke risk assessment [11, 27]. EFT volume also enhances conventional risk models; when added to the CHA_2_DS_2_‐VA score, it improves stroke prediction, particularly in intermediate‐risk groups [28]. EFT volume is a critical biomarker linking adipose tissue biology to thromboembolic risk. Established thresholds (e.g., > 4.05 mm for anticoagulation decisions and > 112 cm^3^ for post‐thrombolysis risk) facilitate personalized management, while lifestyle interventions that reduce EFT volume parallel a lower stroke risk.

Our research further clarified that the EFT most closely associated with thrombosis and a hypercoagulable state is localized within 1 mm of the left atrium. This finding suggests that EFT may influence atrial endothelial function through direct contact, potentially leading to a hypercoagulable state and thrombosis. However, further animal studies are needed to verify this hypothesis.

LAAV has emerged as a significant predictor of thrombus formation in patients with AF, supported by evidence from multiple imaging modalities and clinical studies. An enlarged LAAV reflects structural remodeling and hemodynamic dysfunction, creating a prothrombotic environment. For example, Nicol et al. [29] demonstrated that increased LAAV was strongly associated with LAA pseudothrombus, a marker of stasis and stroke risk, even after adjusting for clinical factors such as the CHA_2_DS_2_‐VA score. Similarly, Uziebło‐Życzkowska et al. [30] identified larger LAAV as an independent risk factor for thrombus formation in younger AF patients, highlighting its predictive value across different age groups.

The pathophysiological link between LAAV and thrombogenesis involves reduced blood flow velocity and stasis. Larger LAAVs often correlate with impaired emptying velocity, as demonstrated in studies using TEE and CCTA. For example, Zhao et al. [31] reported that patients with LAAT had significantly higher LAAVs and lower emptying velocities, reinforcing the role of volume‐driven stasis. Additionally, advanced imaging techniques such as cardiac CT have enabled precise volumetric assessments. Kaliyev et al. [32] found that LAAV measurements obtained in the left lateral decubitus position improved thrombus detection, with larger volumes directly associated with thrombus presence.

The prevalence of LAAT observed in our cohort (29.6%) is higher than some literature estimates. This may be related to the single‐center, retrospective design and the specific population studied, which comprised patients scheduled for catheter ablation at a tertiary referral center, potentially selecting for those with more persistent AF, larger atria, or more comorbidities. Furthermore, the inclusion criterion requiring both CCTA and TEE might have enriched the cohort with patients for whom clinicians had a higher pretest suspicion. Therefore, while the identified associations (EFT1 and LAAV) and the model’s discriminative ability remain valid, the absolute risk estimates from our nomogram may not be directly generalizable to an unselected NVAF population and should be validated in broader, prospective cohorts.

Recent evidence underscores the complementary role of circulating biomarkers alongside CCTA metrics for comprehensive risk stratification. For instance, hematological indices such as red cell distribution width (RDW) and cardiac biomarkers like NT‐proBNP have been independently correlated with LAAT in anticoagulated NVAF patients [33]. Additionally, elevated plasma lipoprotein(a) (LP(a)) concentrations have been significantly associated with increased ischemic stroke risk in AF cohorts [34]. Integrating these blood‐based biomarkers with imaging parameters like EFT1 and LAAV may further refine personalized thrombotic risk assessment in future multimarker models.

In summary, CCTA‐based EFT1 and LAAV can be combined with the CHA2DS2‐VA score to develop an effective nomogram prediction model, thereby enhancing the ability to predict thrombosis and hypercoagulable states in patients with nonvalvular AF.

## 5. Limitations

This study has several limitations. First, its retrospective, single‐center design inherently introduces potential selection bias and limits the generalizability of the findings. Second, the predictive model was developed and validated only on an internal cohort; external validation in independent, multicenter populations is necessary to confirm its robustness and clinical applicability. Third, as an observational study, it can identify associations but cannot establish causality between EFT1, LAAV, and thrombus formation. Finally, the model does not incorporate all potential predictors, such as specific biomarkers or detailed echocardiographic parameters of atrial function, which might further improve predictive accuracy. Future prospective studies are needed to address these limitations.

## Funding

This study was supported by Tianjin Health Science and Technology Project, TJWJ2022QN068; Integrated Traditional Chinese and Western Medicine, Tianjin Municipal Health Commission, 12000023P098L0610072U; Tianjin Key Medical Discipline Construction Project, Specialty TJYXZDXK‐3‐017B; and 2025 Clinical Research Funding Program of the Asian Society of Cardiac Electrophysiology, AHRA‐2025‐C‐[0015].

## Conflicts of Interest

The authors declare no conflicts of interest.

## Data Availability

The data that support the findings of this study are available from the corresponding authors upon reasonable request.
